# Fast reproducible identification and large-scale databasing of individual functional cognitive networks

**DOI:** 10.1186/1471-2202-8-91

**Published:** 2007-10-31

**Authors:** Philippe Pinel, Bertrand Thirion, Sébastien Meriaux, Antoinette Jobert, Julien Serres, Denis Le Bihan, Jean-Baptiste Poline, Stanislas Dehaene

**Affiliations:** 1 INSERM U562/ IFR 49, Cognitive Neuroimaging Unit, Gif-sur-Yvette, France; 2 CEA, DSV/I2BM, NeuroSpin Center, Cognitive Neuroimaging Laboratory (LCOG), Gif-sur-Yvette, France; 3 Université Paris-Sud / IFR 49, Cognitive Neuroimaging Laboratory, Gif-sur-Yvette, France; 4 INRIA Futurs, NeuroSpin Center, Computer Assisted Neuroimaging Laboratory (LNAO), Gif-sur-Yvette, France; 5 CEA, DSV/I2BM, NeuroSpin Center, Gif-sur-Yvette, France; 6 CNRS UMR 6152 / Université de la Méditerranée, Laboratoire Mouvement et Perception Marseille, France; 7 Collège de France, Paris, France

## Abstract

**Background:**

Although cognitive processes such as reading and calculation are associated with reproducible cerebral networks, inter-individual variability is considerable. Understanding the origins of this variability will require the elaboration of large multimodal databases compiling behavioral, anatomical, genetic and functional neuroimaging data over hundreds of subjects. With this goal in mind, we designed a simple and fast acquisition procedure based on a 5-minute functional magnetic resonance imaging (fMRI) sequence that can be run as easily and as systematically as an anatomical scan, and is therefore used in every subject undergoing fMRI in our laboratory. This protocol captures the cerebral bases of auditory and visual perception, motor actions, reading, language comprehension and mental calculation at an individual level.

**Results:**

81 subjects were successfully scanned. Before describing inter-individual variability, we demonstrated in the present study the reliability of individual functional data obtained with this short protocol. Considering the anatomical variability, we then needed to correctly describe individual functional networks in a voxel-free space. We applied then non-voxel based methods that automatically extract main features of individual patterns of activation: group analyses performed on these individual data not only converge to those reported with a more conventional voxel-based random effect analysis, but also keep information concerning variance in location and degrees of activation across subjects.

**Conclusion:**

This collection of individual fMRI data will help to describe the cerebral inter-subject variability of the correlates of some language, calculation and sensorimotor tasks. In association with demographic, anatomical, behavioral and genetic data, this protocol will serve as the cornerstone to establish a hybrid database of hundreds of subjects suitable to study the range and causes of variation in the cerebral bases of numerous mental processes.

## Background

Inter-subjects variability is a missing facet of the current neuroimaging literature [1-3], and until recently has been viewed more as a nuisance for brain imaging studies than as a relevant dimension to investigate the mechanisms of human cognition. Indeed, most of the published studies described the cerebral bases of various cognitive processes from voxel-based group analyses performed on the data from 10–15 subjects. Group analysis of a small collection of brains assures that the description of these functional invariants may be extended to other healthy subjects. However we usually do not know if a cerebral network involved in a task is homogenous enough among the healthy population to be analyzed in only one group or if several groups have to be considered, nor how many subjects are required to correctly describe different sub-groups [4] (This question was also recently addressed in [5] on the basis of the present database). Consequently, it is plausible that in many cases, especially in those involving associative areas in complex tasks, we just capture the common denominator of each individual cognitive circuit and lose a large amount of information.

Describing more completely the parts of cerebral networks used but not shared by all of our subjects require considering variability of brain activation, which may have various origins: ▪ *Intra-subject inter-sessions variability *due to movement artifacts, physiological noise, etc... [6,7] ▪ *Spatial variability *caused by the shape and location of cortical sulci [8] even for tasks requiring low-level processing. ▪ *Biological factors *such as sex [9,10], genotype [11-13], or protein expression [14] ▪ *Cognitive skills or difficulties*, which may reflect heterogeneity of the healthy ('control') population of volunteers [15]. ▪ *Cognitive strategies *spontaneously chosen by subjects to perform a task [16-18] or constrained by the protocol [19] ▪ *Education and learning*, that may locally modulate activation or structural anatomy [15,20,21]. Exploring inter-individual variability thus requires investigating various types of co-variation in a multi-dimensional space.

### Toward a multidimensional database

Characterizing this functional variability, particularly when considering the genetic level, ideally requires acquiring functional imaging data from hundreds of subjects and organizing these data into a large-scale database, together with genetic, behavioral and biomorphological data. Databasing and analysis of structural magnetic resonance images has already resulted in probabilistic anatomical atlases [22,23]. However, a similar large scale description of functional networks is still lacking.

Given that we are in the early stages of exploration of the causes of inter-individual variability, it would be desirable for such a functional imaging study to cover a broad variety of cortical territories and to describe cerebral correlates associated with various level of cognitive processing, from simple perceptual processing to higher-level cognitive functions that require explicit learning and education. For instance, recent advances in the genetics of dysphasia, dyslexia, and dyscalculia have provided several candidate genes whose impact on inter-individual variability in the normal population remains unknown. Considering some cognitive tasks that have been extensively described in the neuroimaging literature, we chose to include: a mental calculation task to investigate superior fronto-parietal networks [24] and a language comprehension task which focuses on the inferior frontal and superior temporal lobes [25]. Using auditory and visual stimulation allows us to isolate cortical pathways associated with perceptual processing (superior temporal sulcus and occipito-temporal cortex [26,27]) while the use of conjunction analysis across modalities also allows us to isolate correlates of amodal processing (associative cortices, for instance). Finally, evolutionary and developmental models suggest that some primitive mechanisms may be (partially) shared between hand/finger motor representation, speech language areas and correlates of mental arithmetic [28-30], noticeably in frontal and parietal lobes. If such assumptions are verified, crossing analysis among these tasks may then help to dissect the task-related networks into a more subtle functional parcellation, and enlighten developmental issues of human brain organization. In brief, these considerations suggest that it would be particularly valuable to obtain images of the cerebral substrates of speech comprehension, reading, and calculation in a large number of subjects, associated with genetic, anatomical and behavioral data, in a highly standardized manner, and at a low cost.

**Figure 1:**
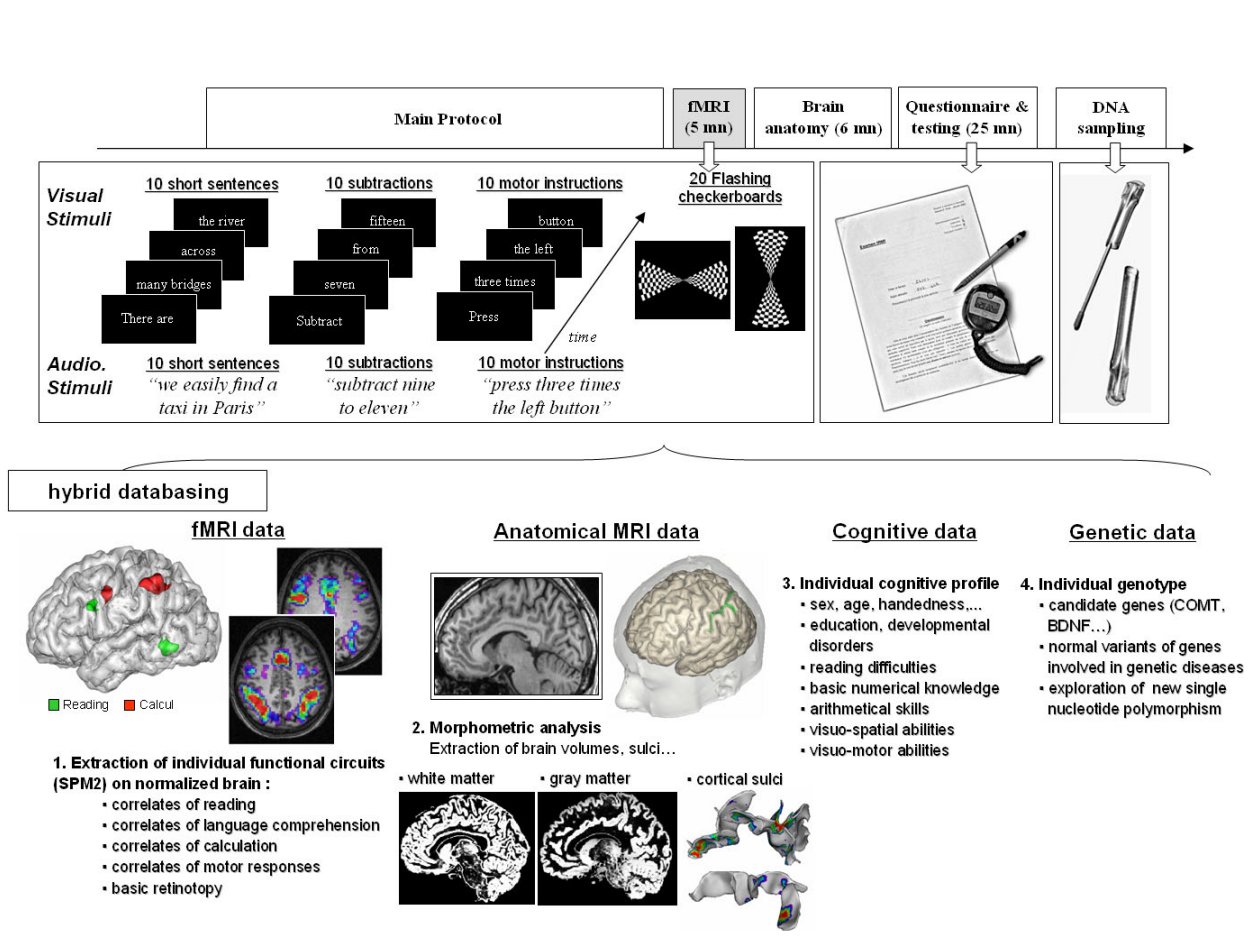
**Summary of data acquisition and databasing**. The top row summarizes the chronology of the functional, anatomical, behavioral and genetic data acquisition. The middle row presents some examples of the tasks used in the fMRI protocol. The bottom row plots summaries of multimodal results which are available for each subject: functional networks related to each experimental condition, anatomical segmentation of grey/white matter and sulci extraction (two left intraparietal sulci are plotted with activation sites for reading and calculation), as well as various behavioral data allowing us to determine a rough cognitive profile of subjects and a genotyping of candidates genes. (Processing and 3D-rendering of brain anatomy were performed using Brainvisa ).

As shown in Figure 1, we planed to acquire four types of data: functional images and a high-resolution anatomical scan for a fine description of sulci, grey and white matter, as well as (not described here) behavioral and personal data, aimed to create a rough cognitive profile of the subject, and DNA sampling (cheek swab). Recall that this data collection occurs within the constraints of this project: to be added to other running protocol, with a minimal cost of people and time.

### A fast brain mapping sequence

In the present research, our goal was to define a simple fMRI test, less than 5 minutes long, that could delineate, in a subject-specific manner, those cerebral circuits. A functional sequence was added to each functional imaging session performed in our lab (Figure 1), taking advantage of the continuous flow of volunteers recruited for various protocols. Because we wanted to capture the maximal amount of functional information in the minimum amount of time, we designed the sequence according the following challenging constraints:

▪ the sequence had to be short, so as to disrupt as little as possible the main protocol. We choose 5 minutes for performing 100 trials.

▪ we aimed to obtain for each subject a description of different levels of functional architecture, from sensori-motor areas (perception and action) to more associative areas involved in reading, language processing and calculation.

▪ we aimed to capture in 5 minutes most of the individual networks related to each task.

▪ individual networks described in 5 min had to be reproducible over sessions and time.

The feasibility of using short stimulation designs (ranging from 10 to 25 min long) to reveal individual functional maps has been previously assessed for language mapping [31], for visual areas [32] and recently for a set of functional networks covering sensorimotor processes, working memory, executive functions and emotional processes [33]. Beyond that point, the main goal of the current study was to use the data obtained with this fMRI protocol with individual subjects as the cornerstone of a large-scale hybrid database. Because the individual functional information that can be captured in such a short sequence should be considered with caution, we focus here on the design efficiency and within-subject. We then describe preliminary data obtained from 81 subjects scanned in a 3T scanner and address new methodological issues including statistical methods for analysis and visualization of inter-individual functional variability. Subsequent publications will exploit the potential of this database to focus on characterizing inter-individual variability.

## Results

### Individual information captured in 5 minutes

**Figure 2:**
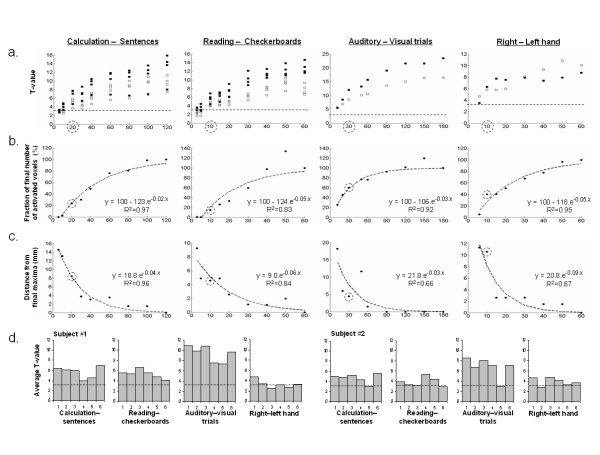
**Impact of the trials number onto the description of individual functional maps**. We plotted the evolution of several descriptors of cerebral activation with the number of trials acquired. A dotted circle indicates the value or number of trials obtained after 5 minutes of acquisition. a) Evolution of the t-value with the number of trials performed for 4 peaks selected for 4 contrasts plotted for 2 subjects (black square = subject 1, white square = subject 2). The dotted line represents the t-value corresponding to a voxel p value < 10^-3^). b) Number of activated voxels in the whole brain (p < 0.001 uncorrected, minimum cluster extent of 10 voxels) relative to the final number of voxels activated after 6 blocks (averaged over subjects). The dotted curve represents the fitted logarithmic curve. c) Evolution of the distance (mm) of selected peaks from their respective final location after 6 blocks (averaged over subjects). The dotted curve represents the fitted logarithmic curve. d) For each subject and each contrast the mean t-value of the 500 most significant voxels for each of the 6 blocks is plotted. The line represents t-value corresponding to a voxel p value < 10^-3^.

Figure 2 shows the evolution of the statistics of four functional contrasts of two subjects with scanning time (T value, number of activated voxels, spatial location), allowing us to determine the minimal number of blocks required to reach a satisfactory description of the individual maps. Interestingly, all of the selected peaks exceeded the p < 0.001 threshold at voxel level after only one block and averaging t-values showed that one block was enough to reach about 60% of the final statistical significance. Locations of these peaks were on average 7 mm away from the final coordinates obtained after completion of 6 blocks. Finally, we reported that mean intra-subject and intra-session activation significance varies with block and across contrasts, suggesting that weaker activation peaks may be missing in some blocks when a fixed statistical threshold is applied. Importantly, this variability does not seem to reflect overall factors such as arousal or attention, which would have been predicted to yield similar profiles of activation for each contrast across sessions.

**Figure 3:**
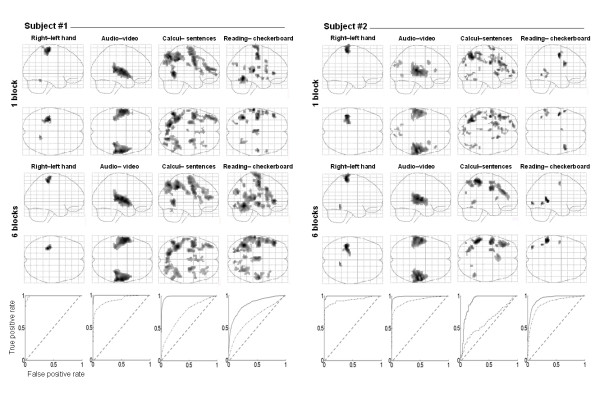
**Individual functional information captured in 5 min**. Illustration of functional information captured in one 5-minute session (one block) compared with a 30-minutes long session (6 blocks), for two subjects. Individual correlates of four tasks are plotted (sagital and axial view) at p < 10^-3 ^uncorrected (cluster extent 10 voxels) for the one block analysis. Similar correlates are plotted at p < 10^-3 ^corrected (cluster extent 10 voxel) for the six block analysis. Below are plotted ROC curves of each subject's contrast images. Solid line represent curve obtained using the t-value map of the corresponding subject, while dotted line represent curve obtained using the t-value map of the same contrast but of the other subject. Diagonal lines represent the ROC curves that would be obtained in the case of a non-informative random map.

Reliability of individual maps obtained from one single block is assessed in Figure 3. Functional networks obtained from the first 5-minutes block appeared very similar to those obtained after a 30-minute session, except that the statistical significance increased with the number of blocks performed, thus allowing for a more stringent threshold. A first threshold-dependant quantitative approach, detailed in Table 1, showed that about the three-fourths of maxima reported for the two subjects after the completion of 6 blocks were already present as local or main maxima in the one-block analysis, with an averaged spatial shift of 6 mm (2 voxels), varying between 2 and 10 millimeters depending of subject and task. Conversely, about 40% of maxima isolated in the one block analysis did not appear as a local/main maxima in the six blocks analysis at the selected threshold. More interestingly, a second threshold-free approach (ROC analysis, Figure 3) revealed a high probability of a consistent classification of voxels into active and inactive categories across the 5-minutes and 30-minutes maps of a subject. For right hand action, auditory stimulation, calculation and reading task, respectively, the discriminative power (D_p_) values were 1, 0.99, 0.98 and 0.87 for the first subject and 0.99, 0.99, 0.95 and 0.94 for the second one. We also reported a gradient of subject-specificity over contrasts: for right hand action, auditory stimulation, calculation and reading task, respectively, the averaged inter-subject D_p _values were 0.95, 0.92, 0.78 and 0.63. This result confirms the sufficiency of the 5-minutes map and suggests high intra-subject consistency but lower cross-subject reliability for reading and calculation tasks.

**Table 1:** Concordance between activation peaks listings reported from 5 min (p < 10^-3 ^uncorrected, cluster extent 10 voxels) or from 30 minutes (p < 10^-3 ^corrected, cluster extent 10 voxels) of functional acquisition, for four contrasts and 2 subjects

	**right-left hand action**		**audio – video trials**		**reading – checkerboard**		**calculation – sentences**		
	
	subject 1	subject 2	subject 1	subject 2	subject 1	subject 2	subject 1	subject 2	*mean*
▪ proportion of the 6 blocks session local maxima detected as local maxima of the first block	100% (of 2 max.)	67% (of 3 max.)	100% (of 8 max.)	82% (of 11 max.)	54% (of 41 max.)	80% (of 5 max.)	83% (of 35 max.)	84% (of 19 max.)	***74% ****(of 124 max.)*
▪ proportion of the 6 blocks session main maxima detected as main maxima of the first block	100% (of 2 max.)	67% (of 3 max.)	80% (of 5 max.)	80% (of 5 max.)	82% (of 11 max.)	80% (of 5 max.)	70% (of 10 max.)	82% (of 11 max.)	***79% ****(of 52 max.)*
▪ proportion of the first block local maxima not detected as local maxima of the six blocks session	50% (of 4 max.)	0% (of 2 max.)	11% (of 9 max.)	50% (of 18 max.)	4% (of 23 max.)	55% (of 9 max.)	29% (of 38 max.)	62% (of 43 max.)	***38% ****(of 146 max.)*
▪ proportion of the first block main maxima not detected as main maxima of the six blocks session	50% (of 4 max.)	0% (of 2 max.)	20% (of 5 max.)	20% (of 5 max.)	18% (of 11 max.)	55% (of 9 max.)	41% (of 12 max.)	62% (of 24 max.)	***41% ****(of 146 max.)*
▪ mean distance for local maxima detected in the first block from their final coordinates reported in the 6 blocks session	9 mm	11 mm	5 mm	4 mm	4 mm	4 mm	5 mm	10 mm	***6 mm***
▪ mean distance for main maxima detected in the first block from their final coordinates reported in the 6 blocks session	9 mm	11 mm	3 mm	3 mm	2 mm	4 mm	5 mm	10 mm	***5 mm***

The first two lines describe respectively the number of maxima successfully isolated with the short 5 min brain mapping and the proportion of maxima reported in the first block but not in the six blocks session. The third line gives an estimation of the spatial precision of peak location after one block (calculated as the average distance from the location obtained after 6 blocks). Analysis were performed both for 'local' maxima (all maxima listed in SPM at that threshold) and 'main' maxima (most significantly activated peaks – see methods).

Interestingly we directly compared individual activation maps obtained from the present protocol with those obtained from a more classic block-design paradigm completed during the same session. In the Additional File 1 we show six examples of within-subject reproducibility across experimental designs. These results suggest that the main individual foci of activation collected in our database truly reflect areas crucial for a cognitive task independently of the design (block vs. task shifting), number of trials and task details.

### Inter-/intra-subject variability

**Figure 4:**
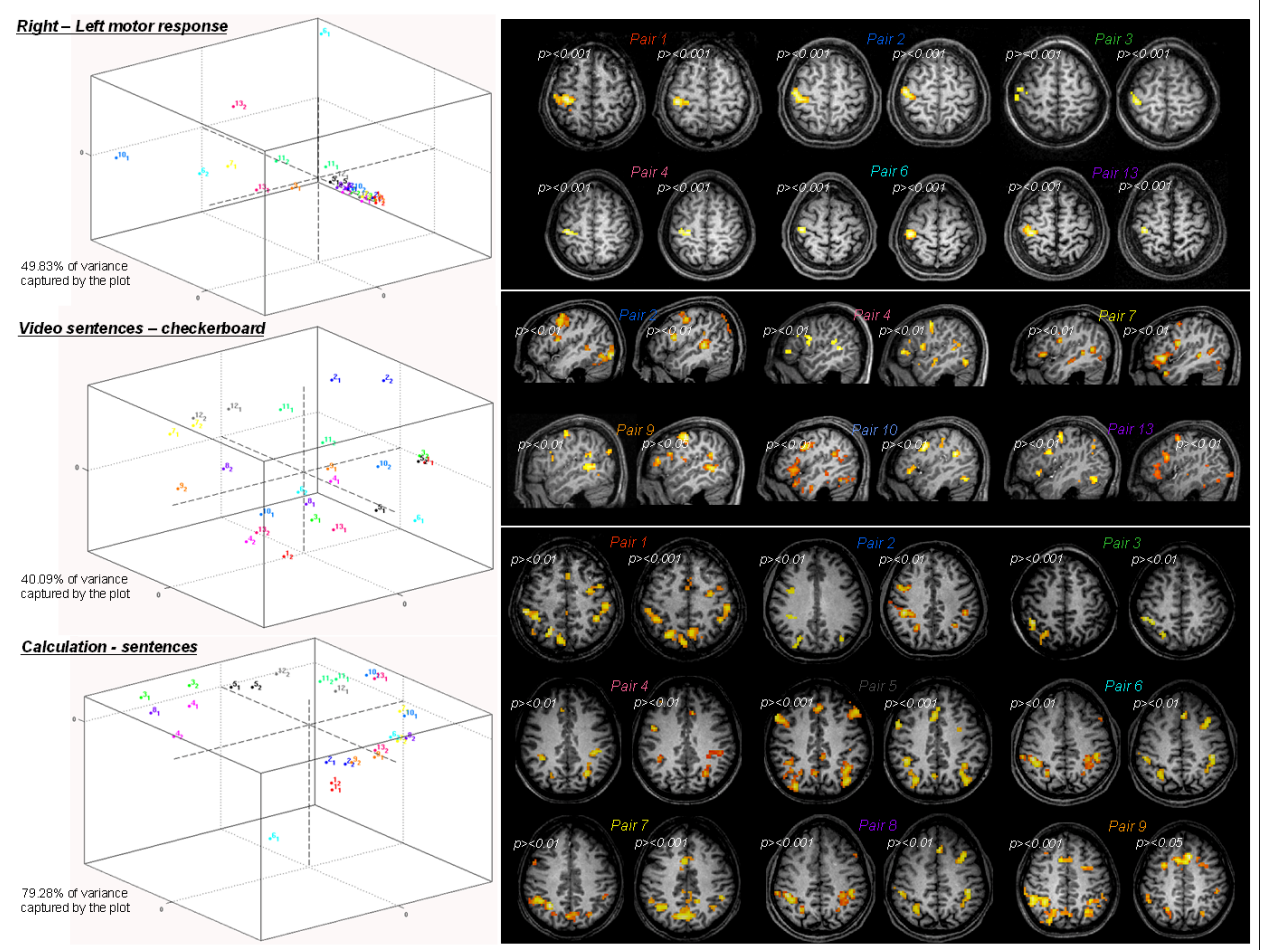
**Inter-subjects and inter-sessions within-subject variability**. For three contrast, a MDS representation of inter-session distance is plotted (left side) in the 3D space which captured most of the variance. Different dot colors correspond to different subjects, and same color dots correspond to the different sessions performed by the same subject. On the right side, this variability is illustrated by individual statistical maps, projected on an axial slice for motor contrast (motor cortex), left sagital slice (covering superior temporal and middle frontal gyri) for reading and an axial slice for mental calculation (intra-parietal region). Each pair refers to two sessions of the same subject (session 1 plotted to the left of session 2) and colors correspond to the dot colors in the MDS graphs. Threshold level was adapted for each session (p < 10^-2 ^or p < 10^-3^uncorrected at voxel level) to enhance topology similarity for pair sessions.

Examination of individual representative contrast images from subjects who participated in two fMRI sessions often indicated a high reliability of the activations (Figure 4, right column). Inter-subject variability is also illustrated and appears to vary with task: for the motor contrast, subjects displayed a similar pattern of activation around the left central sulcus. For the reading contrast, the inter-subject variability seems much higher, due to various combinations of activation, but mainly restricted to the left hemisphere (due to language left-lateralization). For calculation, spatial variability across individuals extended both in lateralization and in the relative amounts of frontal versus parietal activity. For instance, subject 5 strongly activated bilateral fronto-parietal regions, but only a subpart of this pattern was isolated for subject 2 and 3.

To quantify these observations, an inter-scans distance based on the calculation of a similarity coefficient were computed between activation patterns of the 13 subjects and 2 sessions and plotted in a reduced 3-dimensional space for three contrasts (Figure 4). A high degree of within-subject reliability was observed for reading and calculation tasks, as the two scans obtained in a given subject (dots with same color) were mostly tightly grouped together in this summary space. Considering the small size of the motor activations, we suspected that the distance computation was strongly influenced by voxels of non-interest. Indeed, when we masked the distance computation by the right-left motor activation from the RFX group analysis, all pairs of intra-subject scans were grouped together and the variance explained increased up to 66%. Overall, this suggests a high degree of similarity between intra-subject scans both in location and time course of activations. This observation was statistically assessed by a non-parametric Wilcoxon signed rank test computed on the 13 differences between inter-scan intra-subject distance (scan 1 – scan 2 distance) and averaged inter-scan inter-subject distances (scan 1 – 24 other subject's scans). P values for the difference between intra and inter-subject distances were 0.0215, 0.0398 and 0.0012 for motor, reading and calculation task, respectively.

### Group analysis of main cerebral networks

**Figure 5:**
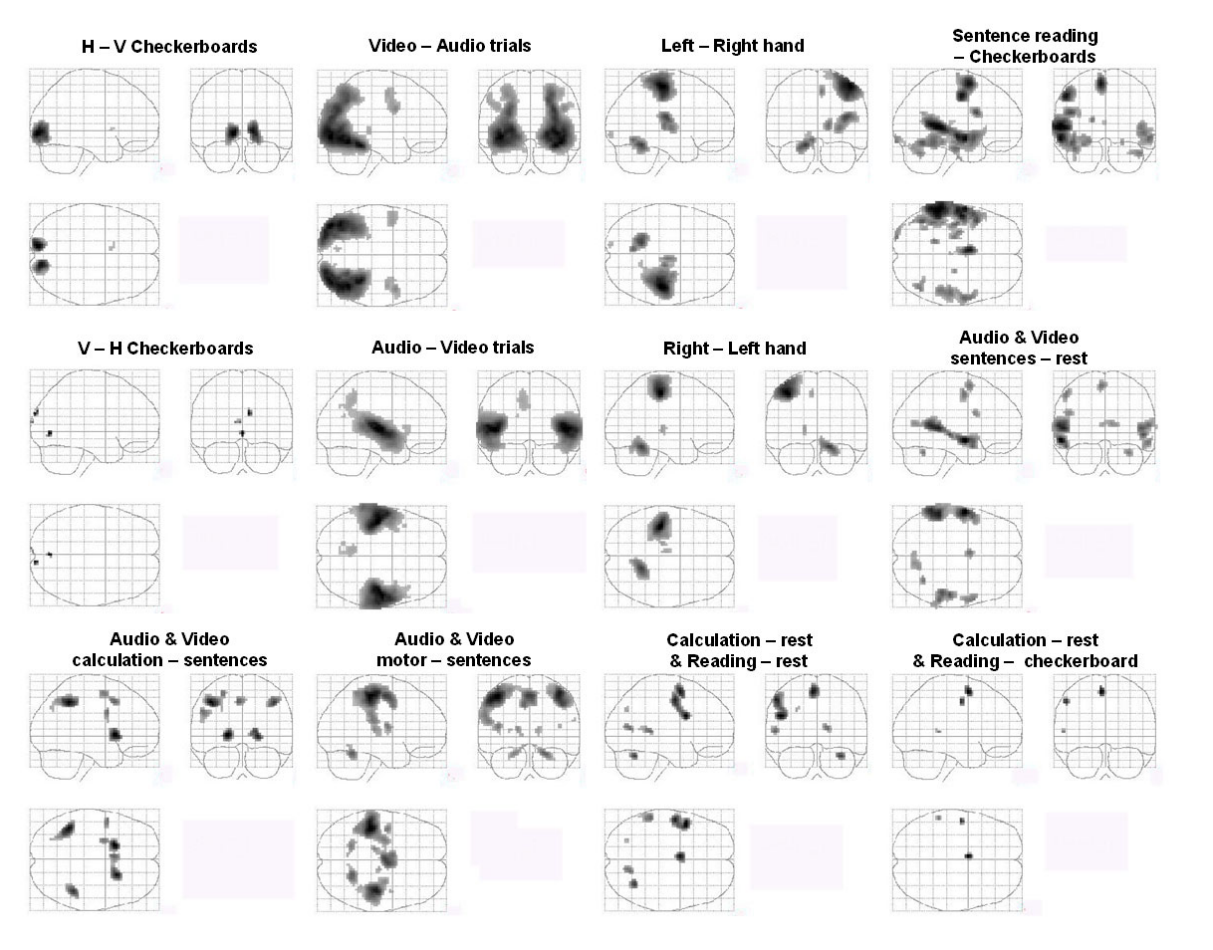
**RFX group analysis**. Contrast images of sensorimotor processes, word reading, native language encoding and mental calculation, shown on SPM glass-brains (sagital, coronal and axial view). Random effect analyses were performed on 81 subjects (p < 0.05 corrected for multiple comparison across brain volume, with a minimal cluster extent of 5 voxels).

A voxel-based random effect analysis (RFX) performed on the 81 subjects' contrast images allowed us to compare the efficiency of our fast protocol with results already described in the neuroimaging literature (see Figure 5 and Table 2 for a detailed listing of areas – maxima were labeled using the Anatomical Automatic Labeling (AAL) program package [34] except for visual areas where a functional labeling was preferred). Contrasts based on checkerboards, auditory and visual stimuli activate cortical areas related to perception and stimulus encoding: occipital lobe, several sites of the occipito-temporal pathway, auditory temporal cortex. Left and right hand action contrasts demonstrated activation of contralateral sensorimotor cortex, SMA and ipsilateral cerebellum, while the conjunction of audio and visual motor – sentences contrasts showed a large bilateral set of areas included SMA, rolandic operculum, cerebellum, thalami, postcentral and precentral areas. The main correlates of reading were a set of middle and superior temporal areas with a strong trend toward left lateralization, left frontal sites and inferotemporal areas and a set of subcortical sites. The conjunction of auditory and visual sentences mainly restricted the previous network to bilateral middle and superior temporal sites that surround auditory cortex and left frontal areas. The conjunction of audio and visual calculation minus sentences showed increased activation in bilateral intraparietal areas, putamen and left precentral gyrus. Because it is supposed that the cerebral mechanisms of mental numerical manipulation may partially overlap with visuospatial and language processing, we isolated areas common to calculation and reading: the conjunction of auditory, visual calculation and reading versus checkerboards isolated a small left lateralized network comprising SMA, precentral and temporal area. The conjunction of auditory, visual calculation and reading versus rest revealed additional left frontal and superior occipital sites, right cerebellum and right calcarine activation.

**Table 2:** Brain areas activated for each condition and conjunction displayed on figure 5

	**Coordinates**			
		
**Brain area**	**x**	**y**	**z**	**T value**
***Horizontal – Vertical checkerboards***				
L V1 area	-12	-90	-6	13.27
R V1 area	15	-84	-3	11.19
Left pallidum	-15	9	0	10.68
***Vertical – Horizontal checkerboards***				
V1/V2v border	0	-75	-3	6.32
V3/V3A border	9	-93	24	5.87
V1/V2d border	-3	-96	12	5.77
***Audio trials – visual trials***				
L Heschl gyrus	-45	-18	3	25.07
R Heschl gyrus	48	-15	6	24.46
L sup. temporal gyrus	-57	-6	0	20.93
R sup. temporal gyrus	60	-18	3	20.81
R sup. temporal gyrus	54	-6	-3	20.29
L sup. temporal gyrus	-66	-39	9	14.14
***Video trials – Audio trials***				
L inf. occipital gyrus	-39	-84	-6	19.79
R fusiform gyrus	42	-60	-12	18.84
R mid. occipital gyrus	36	-81	-3	18.56
R fusiform gyrus	33	-42	-18	18.52
L inf. temporal gyrus	-42	-69	-9	18.40
L mid. occipital gyrus	-30	-90	0	16.99
R precentral gyrus	45	3	30	9.31
R precentral gyrus	39	-6	51	7.40
L precentral gyrus	-45	0	33	7.24
L hippocampus	-24	-33	0	6.61
***Left motor – Right motor***				
R postcentral gyrus	45	-24	60	15.64.21
L cerebelum	-15	-51	-18	11.88
R rolandic operculum	39	-21	18	11.42
L cerebelum	-6	-63	-12	9.87
R thalamus	15	-21	9	9.32
L lingual gyrus	-12	-93	-6	6.23
R putamen	30	-12	3	9.07
R supp. motor area	6	-18	51	9.03
R precuneus	12	-51	72	5.75
R paracentral lobule	12	-18	78	5.50
***Right motor – Left motor***				
L poscentral gyrus	-39	-27	54	17.46
R cerebelum	15	-51	-18	12.85
L thalamus	-18	-24	6	7.08
L supp. motor area	-6	-18	51	6.81
***Motor – Sentence trials***				
L postcentral gyrus	-42	-30	54	14.19
R precentral gyrus	39	-21	57	12.68
supp. motor area	0	-9	54	10.90
R cerebelum	18	-54	-21	9.51
L supp. motor area	-6	0	45	8.98
L rolandic operculum	-42	-6	12	8.44
L cerebelum	-18	-54	-21	8.06
L thalamus	-15	-24	9	7.61
R precentral gyrus	30	-9	60	7.41
R thalamus	12	-18	9	6.51
R rolandic operculum	42	-3	12	6.26
L cerebelum (vermis)	-6	-60	-12	5.97
R inf. parietal gyrus	36	-42	48	5.97
R postcentral gyrus	60	-21	21	5.88
L putamen	-27	-6	9	5.64
R cerebelum	9	-57	-12	5.42
***Sentences reading – checkerboards***				
L mid. temporal gyrus	-57	-39	6	11.54
L sup. temporal gyrus	-60	-3	-12	10.95
L precentral gyrus	-51	-9	45	10.72
L supp. motor area	-6	0	63	10.64
L supp. temporal gyrus	-57	-12	-9	10.36
L inf. frontal gyrus (tri.)	-45	12	24	8.82
R sup. temporal gyrus	57	-6	-9	8.01
R mid. temporal gyrus	51	-24	-3	7.81
L precuneus gyrus	-18	-45	15	7.48
R mid. temporal gyrus	48	-36	3	7.47
R cerebelum	45	-63	-30	7.41
L lingual gyrus	-21	-96	-15	7.4
L inf. temporal gyrus	-45	-60	-15	7.17
L fusiform gyrus	-39	-15	-24	6.76
R hippocampus	24	-18	-15	6.61
L hippocampus	-24	-18	-15	6.6
R cerebelum	36	-60	-30	6.45
L angular gyrus	-33	-57	21	6.33
Thalamus	0	-24	9	6.33
L lingual gyrus	-39	-84	-18	5.33
***Auditory & visual sentences***				
L sup. temporal gyrus.	-57	-3	-9	12.46
L mid. temporal gyrus	-54	-39	6	11.09
L mid. temporal gyrus	-60	-48	9	10.77
R mid. temporal gyrus	51	-24	-3	9.23
R sup. temporal gyrus.	48	-33	6	8.86
R mid. temporal gyrus	57	-39	9	8.41
L mid. frontal gyrus	-51	-3	51	7.83
L supp motor area	-6	3	63	7.78
R sup. temporal gyrus	57	6	-15	7.74
R cerebelum	33	-60	-27	7.67
L inf. frontal gyrus (oper.)	-48	9	27	7.10
R calcarine	9	-69	12	6.86
L calcarine	-3	-72	12	6.08
***Calculation – Sentences***				
L putamen	-18	12	0	9.60
L inf. parietal gyrus	-42	-48	45	9.34
R putamen	21	15	0	9.10
R inf. parietal gyrus	42	-42	45	8.27
ant. cingulate gyrus	-3	12	45	7.10
L precentral gyrus	-51	3	30	6.83
L sup. frontal gyrus	-27	0	57	6.60
L sup. occipital gyrus	-27	-72	39	5.53
***Calculation – rest & Reading – rest***				
L inf. frontal gyrus (oper.)	-48	9	27	9.75
L precentral gyrus	-54	-6	48	8.83
L supp. motor area	-6	0	60	9.12
R cerebelum	33	-60	-27	8.07
L mid. temporal gyrus	-54	-39	6	7.19
R calcarine	15	-66	9	7.12
L sup. occipital gyrus	-27	-69	33	5.76
***Calculation – rest & Reading – checkerboard***				
L supp motor area	-6	0	60	7.57
L precentral gyrus	-51	-9	45	6.77
L mid. temporal gyrus	-54	-39	6	5.91

For each contrast, areas were listed (R = right, L = left) with their spatial coordinates in stereotaxic space (MNI) and t-value from SPM random effect analysis on 81 subjects (p < 0.05 corrected for multiple comparison across brain volume, with a minimal cluster extent of 5 voxels)

### Preliminary database exploitation

**Figure 6:**
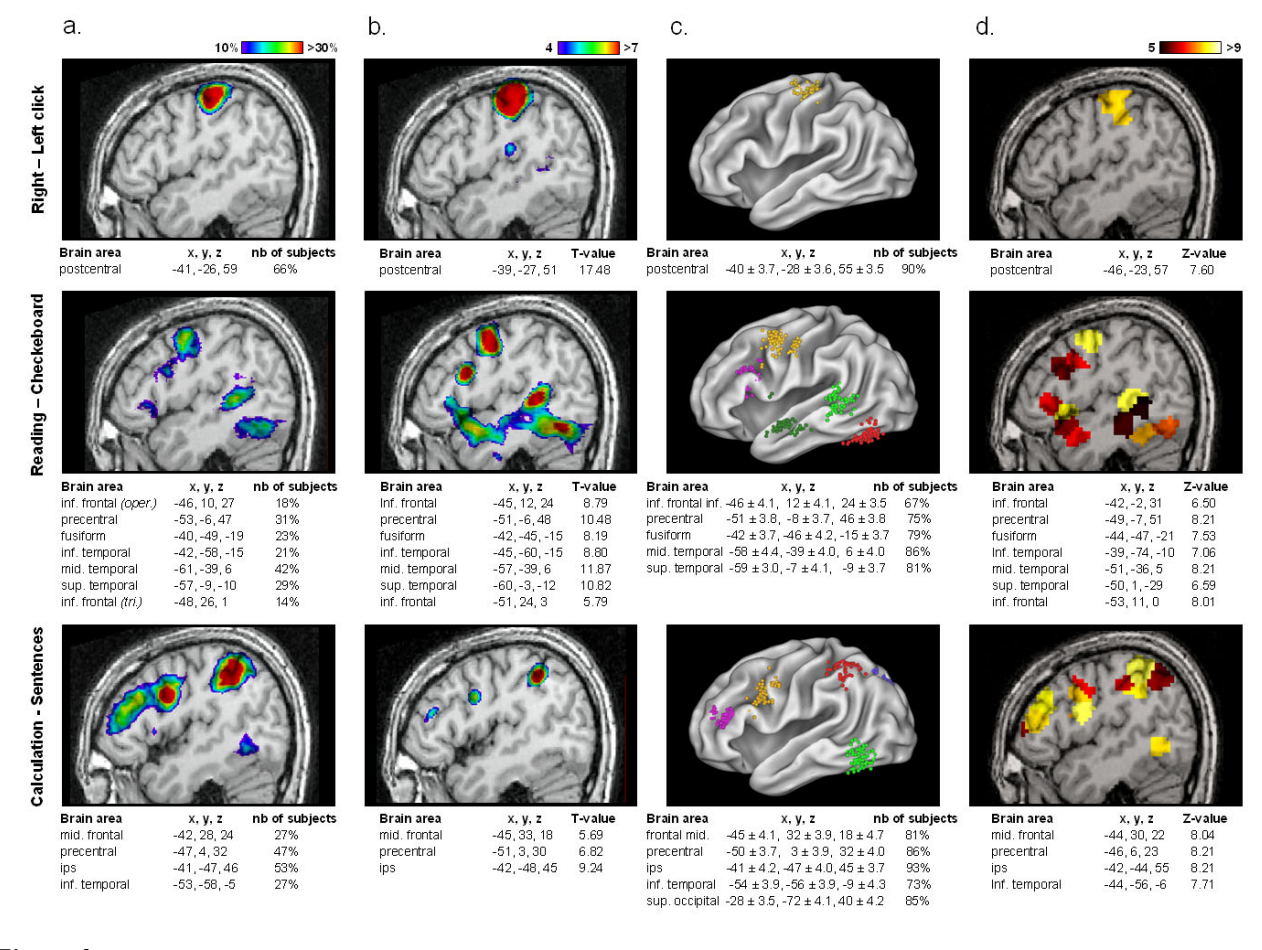
**Comparison and convergence of multiple group analyses**. We compared voxel-based and non voxel-based group analyses for three contrasts and displayed results for the left hemisphere (one individual left sagital slice): right motor activation (first row), reading-related activation (second raw) and left calculation network (third raw). a) RFX map thresholded at Z > 4. b) Overlap of individual statistical maps thresholded each at p < 10^-2 ^uncorrected. Color scale ranges from 10% to 30% overlap. c) Brain functional landmarks of the group, plotted on an inflated human template brain (with Caret software , Van Essen et al., 2001). Color code is used to mark all individual maxima corresponding to one functional area identified in the RFX analysis. Motor activation; yellow = central sulcus. Reading; yellow = precentral gyrus, purple = inf. frontal gyrus, light green = mid. temporal sulcus, dark green = sup. temporal sulcus, red = fusiform gyrus. Calculation; yellow = precentral gyrus, purple = mid. frontal gyrus, red = intraparietal sulcus, blue = sup. occipital gyrus, light green = inf. temporal gyrus. Number of subjects correspond to the number of BFLs with a p < 10^-2^. d) Parcel-based RFX map thresholded at Z > 5. Largest significances for each contrast are colored in yellow (coordinates of each cerebral area correspond to the centre of the most significantly activated local parcel). (*abbreviation: ips = intraparietal sulcus, oper. = pars opercularis of inf. frontal cortex, tri. = pars triangularis of inf. frontal cortex*).

**Figure 7:**
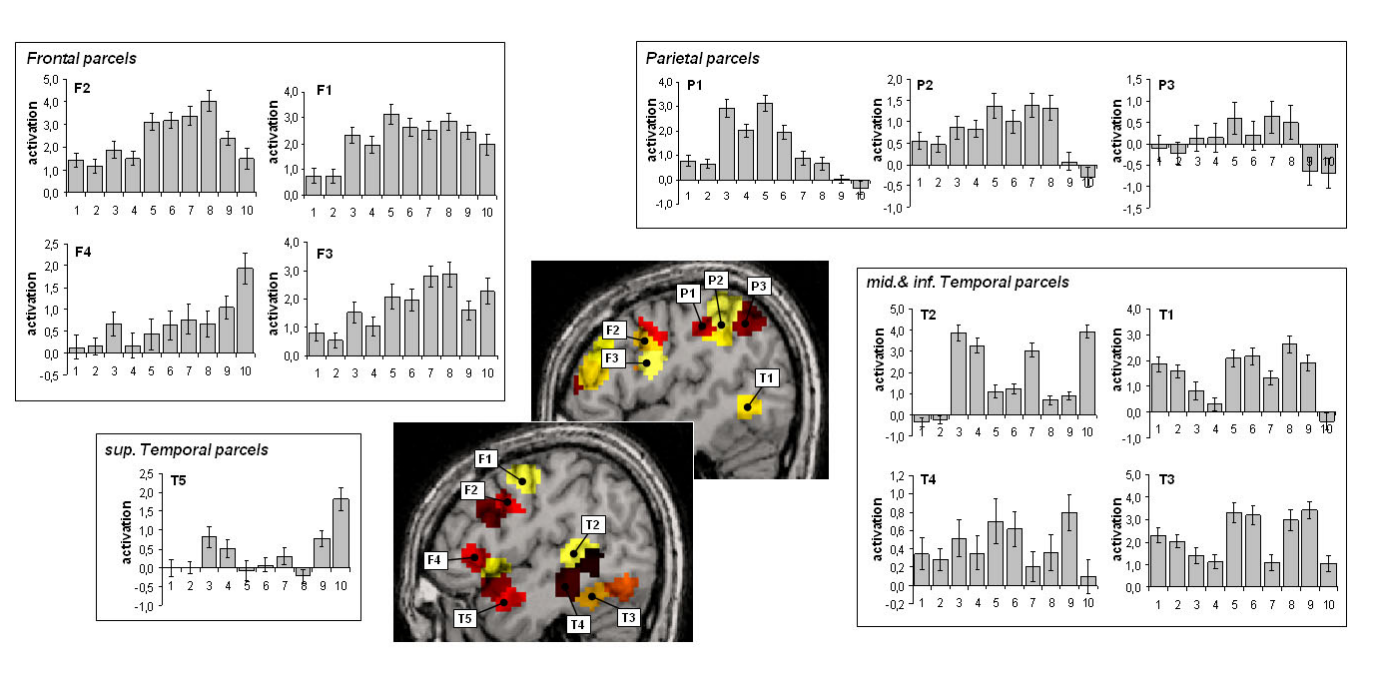
Functional profiles of parcels. We detailed parcels of the left hemisphere for their significant activation (Z > 5) in the reading task (lower central sagital slice) and in the calculation task (upper slice). Histograms represent activation amplitude (%) across the ten tasks, averaged over subjects and plotted for 4 frontal, 3 parietal and 5 temporal parcels. Numerical code for the task is: 1 = horizontal checkerboard, 2 = vertical checkerboard, 3 = auditory right press command, 4 = auditory left press command, 5 = visual right press command, 6 = visual left press command, 7 = audio calculation, 8 = video calculation, 9 = video sentences, 10 = audio sentences.

We compared different approaches for the functional group analysis to test methods that should both improve the statistical power of the group analysis and automatically collect individual functional information of a large fMRI database. A set of highly significant temporal, frontal and parietal activations related to motor, reading and calculation processing were isolated with the voxel-based RFX and plotted on a left sagital slice (column 6a). However, the overlapping of individual functional maps thresholded at p < 10^-2 ^at the voxel level shows that the activated voxels are rarely common to more than a third of the subjects (Figure 6b). Even in left motor cortex, the highest degree of overlap was about 66%. Conversely, some areas, like the left inferior temporal gyrus in the calculation contrast, were present in overlap maps but did not pass the RFX threshold. We enhanced the level of cross-subjects replication when using non-voxel based group analyses. The first one aimed to extract automatically for each contrast a list of individual maxima (called functional landmarks, or BFL) reliable across subjects but not located at exactly identical coordinates that conveys subject-specific information of activation magnitude, statistical significance and spatial location. Detection of single-subject peaks then reached 90% in left motor cortex and 93% in left intraparietal area (column 6c), that are supposed to be crucial for motor and calculation tasks, respectively. Interestingly, the average coordinates of the BFLs were close to the corresponding peak locations found in the overlap and RFX maps. The variances of BFLs location were comparable across all contrasts: the standard deviation error for Talairach coordinates was about 3.5–4 mm for most of the functional landmarks located in both sensori-motor and associative areas. A second procedure was performed to identify similar brain areas (parcel) across subjects mainly on the basis of their functional profile across conditions (Figure 6d). This functional parcellation isolated networks with a very similar topology to those observed with RFX analysis, but with an increase of sensitivity and more extended regions of activation functionally dissociated in subsets of areas: in Figure 7, we report in detail the functional profiles for some of these parcels. It appeared that the parietal activations reported in the voxel-based RFX correspond to a functional gradient, with an anterior site (area P1) strongly involved in all motor trials, the horizontal part of the IPS (P2) equally involved in motor action and calculation, while motor activation was essentially absent in the posterior intraparietal sulcus (P3) which seems more specifically activated by calculation trials, and shows a significant deactivation for language processing. Similar mosaics can be described in frontal lobe: descending from upper part of the precentral gyrus to inferior frontal sites, we observe an area strongly activated for all tasks except checkerboard flashing (parcel F1), then an area which shows preferential activation for motor and calculation tasks (F2). Below this (F3), preference for auditory sentence increases to reach its maximum specificity in (F4) close to Broca's area, but also in the anterior part of the superior temporal gyrus (T5). The posterior superior temporal areas show a high sensitivity to auditory material compared to visual ones (T2) whereas this tendency is inverted in a very close middle temporal area (T1) and reaches its maximal preference for visual word perception in the fusiform gyrus (T3).

## Discussion

Designing a functional database of hundreds of healthy brains while minimizing time and resources costs was the original question addressed by the present work. Here, we present an approach based on the accumulation of homogeneous fMRI data (in combination with behavioral and genetic data) from a sample of a hundred of subjects in a very brief standardized cognitive protocol.

### Efficiency of the fast multi-functional localizer

Protocol duration was the main constraint in the current project. Adding a 5-minute functional scan to each fMRI acquisition performed in the lab could be done without difficulty. Our results indicate that this was sufficient to describe in a subject-specific and reliable way individual topologies of brain activations, with an average spatial accuracy of 2 voxels.

Furthermore, when several subjects were tested twice at an interval of several months, the topology of activations was largely reproducible, even for cognitive tasks such as mental calculation which might be expected to be subject to fluctuations due to learning or attention.

It is worth mentioning that for some subjects, similar patterns were obtained for the two sessions only when lowering the threshold of one session. As shown for subjects who performed 6 blocks in one session, the significance level of a given contrast may be subject to fluctuation even during a single session, and considering all subjects' contrasts images at a fixed threshold may obscure similarities and increases the risk of type I or type II errors in maxima detection. This observation is supported by the high discriminative power of individual statistical maps obtained after a 5 min session and strongly argues for a structural description of individual activations [35,36], less dependent on anatomical location and statistical threshold but more consistent over time and sessions.

We emphasize that reproducibility should be assessed relative to our study's goals: as shown in Figures 3 and 4, some activation peaks can be missed by a given 5-minute scan, presumably due to statistical noise, but possibly also due to changes in strategies, attention, or task-related adaptation. In addition, experimental and/or analysis procedures are not infallible as illustrated by a few subjects for whom no BFL was extracted for motor tasks. However, in the present context, which is to create a database intended to perform behavioral-brain correlations at the scale of hundreds of individuals, this level of reliability is likely to suffice.

### Cerebral networks covered by the protocol and cognitive issues

Group analysis performed on a first database of 81 subjects first allowed us to report several functional networks which fit with previous fMRI results in the fields of speech perception, reading, motor execution and number manipulation. Visual stimuli activated the ventral occipito-temporal pathway from basic retinotopic organization to a more anterior site of the left fusiform gyrus involved in visual word processing [37]. Another gradient of functional specialization was observed in superior temporal gyri, from bilateral primary auditory cortices, easily detected with auditory stimulation, up to temporal areas located around the superior temporal sulcus (with a trend toward left hemisphere dominance) and recruited during multimodal sentence comprehension. They closely mirror the speech-processing areas detailed by Price et al. [38], with an anterior temporal activation associated to the processing of sentence structure, and a posterior middle temporal activation close to sites previously associated with sentence-level semantics [39]. We also observed left frontal areas and SMA, which have been already reported for syntactic and semantic processing of sentences [40,41]. The calculation task involved the bilateral intraparietal and fronto-cingular network classically reported as active during simple number manipulation [42-46], together with the bilateral putamen. These subcortical areas are not usually considered as a part of the core numerical system, but have been tentatively linked to sequential processing of multi-stage calculations or to the retrieval of arithmetical facts [44].

In addition to this coarse functional description of brain areas, due to the simplicity of tasks, the co-existence in our protocol of motor, language and calculation tasks allows examination of their respective neural correlates and gives new insights into some debated cognitive issues. For instance, a restricted subset of three left-lateralized cortical areas (precentral gyri, SMA, and posterior middle temporal gyrus) was isolated by examining the conjunction of sentence comprehension and calculation in both visual and auditory modalities. Although studies of patients have suggested a relative semantic and syntactic independence between language and arithmetic in the adult brain, such functional overlap may represent a core system on which language and calculation are articulated regardless of modality. In particular the left posterior temporal gyrus that has been described as a multimodal integration region [47,48] could be a candidate for convergence of visual, verbal and non-verbal magnitude codes. The left superior occipital gyrus and right visual areas, involved in visual stimuli processing (reading task and checkerboard perception), were also equally recruited during calculation trials performed from visual or auditory inputs. This observation is compatible with the hypothesis that processes sustaining mental calculation may involve a top-down activation of a symbolic digital code [45] and may also share some cortical territories with visuo-spatial areas [46]. It was possible to perform a complementary and improved functional dissection of cortical maps using a parcellation technique over experimental conditions and designed to compensate for inter-subjects anatomical differences. For instance, the two bilateral intraparietal clusters isolated in the calculation RFX, while very close to those reported literature, appeared spatially restricted compared to the individual activation maps where more anterior or posterior additional sites can be seen along the intraparietal sulcus. After individual parcellation of functional areas, the parietal areas related to calculation appeared more extended along the intraparietal sulcus, with an antero-posterior functional gradient that corroborates the geometrical layout reported by Simon et al. [49], as well as the hypothesis of distributed overlapping parietal representations proposed by [50]. A detailed examination of the anterior parietal area indicates that a co-location of motor- and calculation-related activations exists at the single subject level, perhaps illustrating an extension of the numerical system to a sensori-motor representation of hands [29]. Interestingly, a recent study of inter-individual variability of the infero-parietal cytoarchitecture showed a reproducible topography of areas that however vary in size and extent [51]. This biological evidence supports the framework of our individual functional parcellation algorithm which assumed, as a methodological constraint, that parcels are connected in a similar spatial organization across brains. However, further investigations have to be done to see if functional parcellation matches is some respects cytoarchitectonic boundaries. This observation is also true for the mosaic of inferior frontal areas, which were underestimated, both in terms of spatial extent and statistical significance by the voxel-based RFX analysis; this lobe appears to have a complex functional topography, with a widespread intermingling of areas involved in motor response, language comprehension and mental calculation. Interestingly, the left inferior temporal area was absent at the selected threshold (Z > 4) for the calculation task voxel-based RFX analysis. Because the functional profile of this parcel shows a consistent involvement in both auditory and visual calculation tasks and because the overlap of activations across different individuals is only about 27%, an elevated degree of anatomical variability may be suspected in this area. In conclusion, the combination of tasks and modalities allows drawing a detailed functional continuum for middle and inferior frontal lobes, middle and inferior temporal lobes and superior parietal lobe, from more modality-dependant areas up to more abstract cortical regions.

### Dealing with anatomical and functional variability at the group level

Even if cerebral correlates of sensori-motor, reading, language comprehension and calculation processes are well identified and reproducible at the group level, subject level analyses revealed that individual activations were distributed in more complex patterns. Here, one of the most variable patterns across subjects, though associated with the highest intra-subject reliability, was the substrate of calculation. All subjects presented several sites of activation in the intraparietal sulci and frontal lobes but their extents, locations and combination varied. Because this individual patterns were highly reproducible over sessions, not only for large but also for small clusters, inter-subject variability should not be considered as noise but as a physiological signature of subject's brain activation during the numerical task. Whether such individual organizations depend on anatomical or functional constraints is a fundamental question. While we still presented our results in the MNI coordinate system and do not take into account the underlying anatomical structures, we believe that the automatic extraction of individual functional landmarks presented here was a first step to estimate for each functionally defined area both realistic spatial variability and frequency in the population and provides a promising context to address this issue. For instance, spatial variability of the left frontal functional landmarks described in our results fits well with the standard deviation associated to normalized left frontal anatomical landmarks location [8], which ranges from 2.5 to 5.7 mm. This underlies how structural changes may directly affect the location of functional sites and matches well with our estimate of their spatial variability. A recent paper reported a similar conclusion considering only visual cortical areas, where spatial variability of sulcal features was found to reflect those of the functional topography [52]. As suggested by Juch et al. [8], automatically extracting and labeling activation and cerebral structures (sulci) jointly at the individual level will help to describe reliable cerebral organizations while separating anatomical and functional sources of inter-individual variability. Interestingly, a preliminary analysis of the spatial distribution of the functional landmarks in the left hemisphere did not show strong differences between tasks, suggesting that higher cognitive circuits are not associated with a greater spatial variability than sensori-motor ones. These results must be viewed with great caution, as this might be due to the algorithm of BFL extraction, which is based on a recursive method primarily derived from the RFX group analysis. However, the range of variability reported here is similar to that reported in another fast functional localizer study [33], at least for left-lateralized language areas (Broadmann area 44/45 and 22/42).

Concerning the functional features that should constrain individual circuits, it is particularly tempting to describe some frontal or parietal associative areas as functional 'nodes' because of their implication in various of tasks. We suspect that these sites are highly relevant to define individual functional reference frames for brain organization, similar to 'sulcal roots' in global brain gyrification [53] that appear early in the fetal brain and seem to constraint the adult global cortical folding. In a similar vein, detection of systematic spatial co-variation/co-lateralization of activation sites with these functionally defined 'nodes' (e.g. between calculation- and language-related areas) may be informative about the links that exist between different cognitive functions, and may help to specify the developmental constraints or evolutionary roots of the functional cerebral networks in the adult brain [54].

### Perspectives for database exploitation

Further analysis and extension of the database to a greater number of subjects will be needed to disentangle the various sources of inter-subject variability listed in the introduction. We estimate that a minimum of 150–200 subjects will be necessary to begin to describe the variety of activation patterns in the population as well as to reach the minimal statistical power required to correctly isolate sub-groups that can be characterized by a combination of behavioral, anatomical, physiological and genetic features. We have no *a priori *assumptions about the rate of these possible 'variants' in the healthy population. For example, we may recall that 5% of right-handed subjects present an absence of usual left-hemispheric dominance in language correlates. Atypical organization of functional networks may also be suspected in subjects with self-reported developmental difficulties in arithmetic (~5% of our 'normal subjects' sample) or in adults who reported suffering of verbal difficulties during childhood (~10% of our sample). Identifying and characterizing some of these variants in various cognitive domains (verbal, visuo-motor, visuo-spatial, numerical...) may require hundreds of subjects. As demonstrated by a few studies, mostly from clinical populations, genetic variation may also contribute to the phenotype of cerebral activation in verbal [55] or numerical cognition [14,56]. A recent work has emphasized that genetic studies of human cognition might greatly benefit from considering a continuum of cognitive abilities in healthy populations [57]. In the further, we plan to explore the impact of genotype on the structural and physiological features of non pathological functional networks, considering the normal range of variation in subjects' verbal, visuo-spatial and numerical abilities.

## Conclusion

We designed a fast multi-functional localizer paradigm that is now routinely applied in our laboratory to isolate, in five minutes, individual cerebral correlates of visual, auditory and sensorimotor processes, reading, language comprehension and mental calculation. We showed here that isolated activation patterns vary noticeably between subjects, especially for more complex cognitive tasks, but are nonetheless replicable between sessions within each subject. Presenting cognitive tasks in different modalities helped to separate distinct areas, and parcellation tools enhanced this functional dissection. In association with the acquisition of brain anatomies, cognitive profile and genotype of subjects, this protocol will serve to establish a hybrid database of hundreds of subjects suitable to study the range and causes of variation in the cerebral bases of numerous mental processes.

## Methods

### Subjects

94 functional scans were acquired: 66 subjects performed one session, 13 subjects were recruited twice with an interval of several weeks, and 2 subjects performed 6 times the protocol in a thirty minutes session. In total, we obtained a database of 81 different functional scans and anatomy of French healthy subjects (35 males and 46 females; 24 ± 4.1 years old; 80 right handed and one left handed according to the Edinburgh inventory), 13 test/re-test situations and 2 multi-sessions to illustrate the evolution of results precision according to the number of scans acquired. All subjects gave their written informed consent. The study was approved by the regional ethical committee (Hopital de Bicêtre, France).

### fMRI experimental design

Ten types of trials were mixed together and presented successively in a fixed order, which appeared random to the subject (Figure 1), with the E-Prime software (Psychology Software Tool, Inc.): 1-passive viewing of flashing horizontal checkerboards (10 trials), 2-passive viewing of flashing vertical checkerboards (10 trials), 3-pressing three times the left button with the left thumb button according to visual instructions (5 trials), 4-pressing three times the right button according to visual instruction (5 trials), 5-pressing three times the left button according to auditory instruction (5 trials), 6-pressing three times the right button according to auditory instruction (5 trials), 7-silently reading short visual sentences (10 trials), 8-listening to short sentences (10 trials), 9-solving silently visual subtraction problems (10 trials), 10-solving silently auditory subtraction problems (10 trials). 20 rest periods (black screen) were inserted in the sequence and served as null event for a better hemodynamic deconvolution. Subjects were briefly instructed before starting acquisition with a visual sequence projected in the scanner during a non-functional acquisition. A post-acquisition debriefing served to ensure that the subject correctly understood and performed the tasks.

Visual stimuli were displayed as four successive screens (250 ms) separated by 100 ms interval, and each composed of group of one to three words, resulting in 1.3 sec of visual stimulation. Auditory stimuli were digitally recorded by a male speaker (resolution of 16 bits and sampling frequency of 22.05 kHz) and had a similar duration (about 1.6 sec for motor instruction, 1.2–1.7 sec for sentences and 1.2–1.3 sec for subtraction). Calculation consisted of a one-digit number (range from 4 to 9) subtracted from a two-digit number (range from 10 to 19). (See Additional File 2 for a detailed description of the stimuli).

The experimental protocol was organized as a fast event-related paradigm. The 100 trials were presented in a fixed sequence with a stochastic SOA (2400 ms, 2700 ms, 3000 ms, 3300 ms or 3600 ms; mean SOA = 3 sec). This sequence was optimized for both statistical detection and hemodynamic response estimation using a Matlab script developed in the lab and inspired by the genetic algorithm of Wager and Nichols 2003 [58]. One original feature of our procedure was to optimize the sequence according to more than one contrast. Eight contrasts of interest were selected: right vs. left hand action, vertical vs. horizontal checkerboards, auditory stimuli vs. rest, visual stimuli vs. rest, auditory calculations vs. auditory non-numerical stimuli, visual calculations vs. visual non-numerical stimuli, auditory stimuli vs. visual stimuli and visual stimuli vs. checkerboards.

### Data acquisition and processing

Functional images were acquired on a 3T Brucker scanner using an EPI sequence (TR = 2400 ms, TE = 30 ms, matrix size = 64 × 64, FOV = 24 cm × 24 cm). Each volume consisted of 34 slices of 4 mm thickness. Anatomical T1 images were acquired with a spatial resolution of 1 × 1 × 1.2 mm. Data were pre-processed using SPM2 software [59] in Matlab7 environment according to the following procedure: slice timing, subject motion estimation and correction by realignment, coregistration of the anatomical image to the MNI template, spatial normalization of functional images (resampled voxel size = 3 × 3 × 3 mm) and smoothing (5 mm FWHM). Each voxel time series was fitted with a linear combination of the canonical hemodynamic response function and its temporal derivative. A temporal high pass filter was applied (cutoff 128 sec. and AR(1) whitening).

Individual contrast images were generated using SPM2. Individual conjunction maps were constructed to isolate voxels that were activated over a fixed threshold for multiple tasks: these maps were created by taking the min value of each of the contrasts considered for each voxel.

#### ▪ Multi-sessions analysis

Two subjects performed the 5 min short protocol six times in a single session (all blocks used the same temporal design but four of the six included new stimuli to partially avoid habituation effects). We performed six SPM2 models, using a third of one block (i.e the first 30% of the trials for each condition of the first block), half of one block, one block, one and half blocks, two blocks, three blocks, four blocks, five blocks and six blocks, respectively. Statistics were collected from various maxima of interest listed from each subject's analysis: two frontal, four parietal and two temporal sites for calculation, four frontal, two inferior temporal, one fusiform and one parietal site for reading, the two left superior temporal maxima for auditory stimuli, and the two left motor area maxima for the right hand action.

For four contrasts, we conducted two SPM2 analyses for each subject using the first experimental block and all six blocks, respectively. Main maxima were listed for both analyses (p < 10^-3^, uncorrected, 10 voxels cluster extent for one block analysis; p < 10^-3^, corrected, 10 voxels cluster extent for the 6 blocks analysis reflecting the increased statistical power with number of trials). For each subject and contrast we calculated the proportion of peaks detected or missed when using only the first block compared to the overall session results, and the precision of their spatial location using Euclidian distance from final coordinates. Because these proportions may be task- and threshold-dependant, we first isolated *local maxima *as all the peaks listed with the SPM2 interface at the threshold noted above, separated by a minimal distance of 8 mm. We then selected as *main maxima *of a functional network peaks greater than two thirds of the highest t-value for the respective contrast.

The threshold-free receiver operating characteristic (ROC) approach compare the power to discriminate overall activated voxels identified by an SPM t-value map generated from trials of a 5 min session with the SPM thresholded t-value map obtained after a 30 min session (p < 10^-3^, corrected, 10 voxels cluster extent). ROC curves represent the plot of the true positive rate (sensitivity) against the false positive rate (1 – specificity) for different thresholds of the 5 min t-value map. The discriminative power (D_p_) was estimated by calculating the area under the curve and may be interpreted as the probability that voxels classified as activated (or non-activated) in the 5 min map were also classified as activated (non-activated) in the 30 min sequence. In addition we calculated an inter-subject D_p _by comparing the 5-min t-map of each subject with the 30-min t-map for the other subject. Similar patterns of activation across subjects would lead to comparable intra- and inter-subject D_p_values.

#### ▪ Intra-/inter-subjects variability

Inter-scan distance were calculated using the SPM2 Distance toolbox [60]. It provides a global and objective measure of the relative 'distance' in time course and pattern of activation between all fMRI sessions for any contrast. A Multi-Dimensional Scaling (MDS) procedure allows a visual representation of the inter-session distance in a reduced 3-dimensional space which captures the greatest proportion of variance.

#### ▪ Group analysis

Various methods of group analysis were applied to the first scan of our group of 81 subjects:

#### Random Effect Analysis (RFX)

Voxel-based RFX analysis were performed on the individual smoothed contrast images and conjunction images (5 mm FWHM) at a voxelwise threshold of p < 0.05 corrected for multiple comparison across the brain volume, with a minimal cluster extent of 5 voxels.

#### Brain Functional Landmark detection

We used an automatic identification method described by Thirion et al. [61]. The list of landmarks is primarily derived from the RFX group analysis thresholded at p < 10^-3^, uncorrected. Reliability of the BFL is based on a recursive leave-one-out procedure and an agglomerative clustering of candidate individual local maxima.

#### Parcellation of fMRI datasets

An automatic parcellation method [62,63] was used allowing up to 10 mm of variability in anatomical location of each parcel. It is based on a spectral clustering algorithm that delineates functionally homogeneous and spatially connected regions over the entire brain. A recent report of Thyreau et al., 2006 [64] suggested 500 parcels per brain as spatial definition. Parcel-based RFX analysis was then performed on the set of individual parcellation, resulting in Z-value maps for each functional contrast.

## Authors' contributions

The project was conceived by PP, JBP and SD. JS was involved in optimization of the paradigm. Data were collected, processed and organized by PP and AJ. Inclusion of volunteers in the protocol was under the responsibility of DLB. Non-voxel based statistical methods were developed by BT, SM and JBP. All authors read and approved the manuscript.

## Supplementary Material

Additional file 1 Comparison of individual activation maps obtained from our 5 minutes protocol with maps computed from fMRI data acquired during the same session with another longer bloc design. As explained in the manuscript, our 5 minute protocol was added to each fMRI session performed in our laboratory. To estimate efficiency of our fast functional mapping and see if its results should be considered as free from its particular design, we compared some of our statistical maps with those corresponding to similar conditions performed in the associated main protocol (see details of each protocol below). We display here two examples of individual contrasts for three different fMRI protocols: one protocol including visual sentence reading (p = 0.001 corrected at the voxel level), and two other protocols including each a calculation task and a verbal control task (p = 0.001 uncorrected at the voxel level). Surrounded by a blue frames are reported a statistical map form the 5 min protocole for the corresponding subject at a similar anatomical location (p = 0.01 uncorrected at the voxel level, 30 voxels for cluster extent). Note that subject 2 performed twice our 5 min protocol with an interval of nine weeks, illustrating reliability of the activation topography. These maps suggest that even with quite different experimental conditions (block design versus fast event-related design, different control tasks, different rate of presentation, sometime different notation, different resolutions, different number of trials...) our 5 min design was able to capture most of the individual cerebral sites that characterize subject's functional activation during a task (here reading or calculating). In conclusion, maxima reported for each subject could be reasonably associated to areas that are crucial to perform a specified cognitive task, independently of the experimental conditions of stimulation and acquisition. Statistical thresholds were adapted for the obvious reason that the statistical power of a protocol depends on the number of trials performed. Experiment 1. Three sessions comprising each 20 miniblocks of 4 visual sentences (total duration = 25 mn/240 sentences). The sentences varied in length from 5 to 17 words, flashed successively at a rate of one word every 270 msec. Subjects had just to read passively sentences and press a button when a probe sentence was displayed (6% of trials). Data acquisition was similar to our protocol (TR = 2.4 s, 24 slices, voxel size = 3 × 3 × 5 mm). Experiment 2. Two sessions comprising each 2 miniblocks of mental calculation and 2 miniblocks of a numerical control task. Each miniblock comprised 9 mental subtractions (total duration = 9 mn/36 trials). Subject had to subtract a visually displayed one Arabic digit number from a memorized reference ('12' for instance) and silently pronounced the result. Numbers was flashed for 200 ms and successively at a rate of one digit every 2200 msec. During the control task, the subject had to pronounce silently the numerical successor of the stimuli ('4' for the stimuli '3'). Data acquisition was close to those of our protocol (TR = 2.4 s, 24 slices, voxel size = 3 × 3 × 5 mm). Experiment 3. One session comprising each 5 miniblocks of mental calculation and 5 miniblocks of a non-numerical control task. Each miniblock comprised 10 mental calculations (total duration = 10 mn/50 mental numerical trials). During mental calculation, subject had to make a serie of subtraction and addition: each visually proposed arithmetical operation (like '+3' or '-5') was applied to the result of the previous calculation. After a serie of five operations, the subject indicated by pressing a button of the final memorized result was similar to the result proposed on the video screen. During the control task, subjects saw a flow of letters. After a serie of five letters, he indicated in a similar way if a visually displayed target letter was present in the previous stimuli. Because a high resolution acquisition was used in this paradigm, parameters of acquisition differed much from our protocol: acquisition was limited to a volume placed on superior frontal and parietal lobes (TR = 3 s, 32 slices, voxel size = 1.5 × 1.5 × 2 mm). Click here for file

Additional file 2 **List of Verbal stimuli**. List of auditory and visually presented stimuli. Stimuli were presented in a French version, but translated for reader and reported here in an italic form. Click here for file

## References

[B1] Seghier ML, Lazeyras F, Pegna AJ, Annoni JM, Zimine I, Mayer E, Michel CM, Khateb A (2004). Variability of fMRI activation during a phonological and semantic language task in healthy subjects. Hum Brain Mapp.

[B2] Mechelli A, Penny WD, Price CJ, Gitelman D, Friston KJ (2002). Effective connectivity and intersubject variability: Using a multisubject network to test differences and commonalities. NeuroImage.

[B3] Brett M, Johnsrude IS, Owen AM (2002). The problem of functional localization in the human brain. Nat Rev Neurosci.

[B4] Friston KJ, Holmes AP, Worsley KJ (1999). How many constitute a study?. NeuroImage.

[B5] Thirion B, Pinel P, Meriaux S, Roche A, Dehaene S, Poline JB (2007). Analysis of a large fMRI cohort: Statistical and methodological issues for group analysis. NeuroImage.

[B6] McGonigle DJ, Howseman AM, Athwal BS, Friston KF, Frackowiak RSJ, Holmes AP (2000). Variability in fMRI: An examination of intersession differences. NeuroImage.

[B7] Smith SM, Beckmann CF, Ramnani N, Woolrich MW, Bannister PR, Jenkinson M, Matthews (2005). Variability in fMRI: a re-examination of inter-session differences. Hum Brain Mapp.

[B8] Juch H, Zimine I, Seghier ML, Lazeyras F, Fasel JHD (2005). Anatomical variability of the lateral frontal lobe surface: implication for intersubject variability in language neuroimaging. NeuroImage.

[B9] Shaywitz BA, Shaywitz SE, Pugh KR, Constable RT, Skudlarski P, Fulbright RK, Bronen RT, Fletcher JM, Shankweiler DP, Katz L, Gore JC (1995). Sex differences in the functional organization of the brain for language. Nature.

[B10] Grön G, Wunderlich AP, Spitzer M, Tomczak R, Riepe MW (2000). Brain activation during human navigation: gender-different neural networks as substrate of performance. Nat Neurosci.

[B11] Hariri AR, Weinberger DR (2003). Imaging genomics. Brit Med Bull.

[B12] Fan J, Fossella J, Sommer T, Wu Y, Posner MI (2003). Mapping the genetic variation of executive attention onto brain activity. Proc Natl Acad Sci USA.

[B13] Egan MF, Goldberg TE, Kolachana BS, Callicott JH, Mazzanti CM, Straub RE, Goldman D, Weinberger DR (2001). Effect of COMT Val108/158 Met genotype on frontal lobe function and risk for schizophrenia. Proc Natl Acad Sci USA.

[B14] Menon V, Kwon H, Eliez S, Taylor AK, Reiss AL (2000). Functional brain activation during cognition is related to FMR1 gene expression. Brain Res.

[B15] Menon V, Rivera SM, White CD, Eliez GH, Glover GH, Reiss AL (2000). Functional optimization of arithmetic processing in perfect performers. Cogn Brain Res.

[B16] Burbaud P, Camus O, Guehl D, Bioulac B, Caillé JM, Allard M (2000). Influence of cognitive strategies on the pattern of cortical activation during mental subtraction A functional imaging study in human subjects. Neurosci Letters.

[B17] Reichle ER, Carpenter PA, Just MA (2000). The neural bases of strategy and skill in sentence-picture verification. Cog Psychol.

[B18] Nadeau SE, Williamson DJ, Crosson B, Gonzalez-Rothi LJ, Heilman KM (1998). Functional imaging: heterogeneity in task strategy and functional anatomy and the case for individual analysis. Neuropsychiatry Neuropsychol Behav Neurol.

[B19] Sohn M, Goode A, Koedinger KR, Stenger VA, Fissel K, Carter CA, Anderson JR (2004). Sex differences in the functional organization of the brain for language. Nat Neurosci.

[B20] Delazer M, Domahs F, Bartha L, Brenneis C, Lochy A, Trieb T, Benke T (2003). Learning complex arithmetic – an fMRI study. Cog Brain Res.

[B21] Dragansky B, Gaser C, Busch V, Schuierer G, Bogdahn U, May A (2004). Changes in grey matter induced by training. Nature.

[B22] Toga AW, Thompson PM, Mega MS, Narr KL, Blanton RE (2001). Probabilistic approaches for atlasing normal and disease-specific brain variability. Anat Embryol.

[B23] Van Essen DC (2002). Windows on the brain: the emerging role of atlases and databases in neuroscience. Curr Opin Neurobiol.

[B24] Simon O, Kherif F, Flandin G, Poline JB, Rivière D, Mangin JF, Le Bihan D, Dehaene S (2004). Automatized clustering and functional geometry of human parietofrontal networks for language, space, and number. NeuroImage.

[B25] Price CJ (2000). The anatomy of language: contributions from functional neuroimaging. J Anat.

[B26] Binder JR, Frost JA, Hammeke TA, Bellgowan PSF, Springer JA, Kaufman JN, Possing ET (2000). Human temporal lobe activation by speech and nonspeech sounds. Cereb Cortex.

[B27] Dehaene S, Cohen L, Sigman M, Vinckier F (2005). The neural code for written words: a proposal. Trends Cogn Sci.

[B28] Rizzolatti G, Arbib MA (1998). Language within our grasp. Trends Neurosci.

[B29] Butterworth B (1999). The Mathematical Brain.

[B30] Gerstmann J (1927). Fingeragnosie und isolierte agraphie; ein neues syndrom. Z Ges Neurol Psychiat.

[B31] Fernandez G, de Greiff A, von Oertzen J, Reuber M, Lun S, Klaver P, Ruhlmann J, Reul J, Elger CE (2001). Language mapping in less than 15 minutes: Real-time functional MRI during routine clinical investigation. NeuroImage.

[B32] Stiers P, Peeters R, Lagae L, Van Hecke P, Sunaert S (2005). Mapping multiple visual areas in the human brain with a short fMRI sequence. NeuroImage.

[B33] Drobyshevsky A, Baumann SB, Schneider W (2006). A rapid fMRI task battery for mapping of visual motor cognitive and emotional function. NeuroImage.

[B34] Tzourio-Mazoyer N, Landeau B, Papathanassiou D, Crivello F, Etard O, Delcroix N, Mazoyer B, Joliot M (2002). Automated anatomical labeling of activations in SPM using a macroscopic anatomical parcellation of the MNI MRI single-subject brain. NeuroImage.

[B35] Coulon O, Mangin JF, Poline JB, Zilbovicius M, Roumenov D, Samson Y, Frouin V, Bloch I (2000). Structural Group Analysis of Functional Activation Maps. NeuroImage.

[B36] Mangin JF, Rivière D, Coulon O, Poupon C, Cachia A, Cointepas Y, Poline JB, Le Bihan D, Régis J, Papadopoulos-Orfanos D (2004). Coordinate-based versus structural approaches to brain image analysis. Artif Intell Med.

[B37] Cohen L, Lehéricy S, Chochon F, Lemer C, Rivaud S, Dehaene S (2002). Language-specific tuning of visual cortex? Functional properties of the Visual Word Form area. Brain.

[B38] Price CJ, Thierry G, Griffiths T (2005). Speech-specific auditory processing: where is it?. Trends Cogn Sci.

[B39] Vanderberg R, Nobre AC, Price CJ (2002). The response of left temporal cortex to sentences. J Cogn Neurosci.

[B40] Friederichi AG, Rüschemeyer SA, Hahne A, Fiebach CJ (2003). The role of left inferior frontal and superior temporal cortex in sentence comprehension: Localizing syntactic and semantic processes. Cereb Cortex.

[B41] Just MA, Carpenter PA, Keller TA, Eddy WF, Thulborn KR (1996). Brain activation modulated by sentence comprehension. Science.

[B42] Chochon F, Cohen L, van de Moortele PF, Dehaene S (1999). Differential contributions of the left and right inferior parietal lobules to number processing. J Cogn Neurosci.

[B43] Dehaene S, Piazza M, Pinel P, Cohen L (2003). Three parietal circuits for number processing. Cogn Neuropsychol.

[B44] Dehaene S, Cohen L (1995). Towards an anatomical and functional model of number processing. Math Cogn.

[B45] Dehaene S (1992). Varieties of numerical abilities. Cognition.

[B46] Hubbard EM, Piazza M, Pinel P, Dehaene S (2005). Interactions between number and space in parietal cortex. Nat Rev Neurosci.

[B47] Calvert GA (2001). Crossmodal processing in the human brain: Insight from functional neuroimaging studies. Cereb Cortex.

[B48] Beauchamp MS, Argall BD, Bodurka J, Duyn JH, Martin A (2004). Unraveling multisensory integration: patchy organization within human STS multisensory cortex. Nat Neurosci.

[B49] Simon O, Mangin JF, Cohen L, Le Bihan D, Dehaene S (2002). Topographical layout of hand, eye, calculation, and language-related areas in the human parietal lobe. Neuron.

[B50] Pinel P, Piazza M, Le Bihan D, Dehaene S (2004). Distributed and overlapping cerebral representations of number size and luminance during comparative judgments. Neuron.

[B51] Caspers S, Geyer S, Schleicher A, Mohlberg H, Amunts K, Zilles K (2006). The human inferior parietal cortex: Cytoarchitectonic parcellation and interindividual variability. NeuroImage.

[B52] Hasnain MK, Fox PT, Woldorff MG (2006). Hemispheric Asymmetry of sulcus-function correspondence: quantization and developmental implications. Hum Brain Mapp.

[B53] Cachia A, Mangin JF, Rivière D, Kherif F, Boddaert N, Andrade A, Papadopoulos-Orfanos D, Poline JB, Bloch I, Zilbovicius M, Sonigo P, Brunelle F, Régis J (2003). A primal sketch of the cortex mean curvature: a morphogenesis based approach to study the variability of the folding patterns. IEEE Trans Med Imag.

[B54] Hasson U, Harel M, Levy I, Malach R (2003). Large-Scale Mirror-Symmetry Organization of Human Occipito-Temporal Object Areas. Neuron.

[B55] Liegeois F, Baldeweg T, Connelly A, Gadian DG, Mishkin M, Vargha-Khadem F (2003). Language fMRI abnormalities associated with FOXP2 gene mutation. Nat Neurosci.

[B56] Molko N, Cachia A, Rivière D, Mangin JF, Bruandet M, Le Bihan D, Cohen L, Dehaene S (2003). Functional and structural alterations of the intraparietal sulcus in a developmental dyscalculia of genetic origin. Neuron.

[B57] Leonard CM, Eckert MA, Kuldau JM (2006). Exploiting human anatomical variability as a link between genome and cognomen. Genes Brain Behav.

[B58] Wager TD, Nichols TE (2003). Optimization of Experimental Design in fMRI: A General Framework Using a Genetic Algorithm. NeuroImage.

[B59] SPM2 Software. http://www.fil.ion.ucl.ac.uk/spm/software/spm2/.

[B60] Kherif F, Poline JB, Mériaux S, Banali H, Flandin G, Brett M (2003). Group analysis in functional neuroimaging: selecting subjects using similarity measures. NeuroImage.

[B61] Thirion B, Pinel P, Poline JB (2005). Finding landmarks in the functional brain: detection and use for group characterization. Med Image Comput Comput Assist Interv Int Conf Med Image Comput Comput Assist Interv.

[B62] Thirion B, Pinel P, Tucholka A, Roche A, Ciuciu P, Mangin JF, Poline JB (2007). Improving sensitivity and reliability of fMRI group studies.. IEEE TMI.

[B63] Thirion B, Flandin G, Pinel P, Roche A, Ciuciu P, Poline JB (2005). Dealing with the shortcomings of spatial normalization: Multi-subject parcellation of fMRI datasets. Hum Brain Mapp.

[B64] Thyreau B, Thirion G, Flandin G, Poline JB (2006). Anatomo-functional description of the brain: a probabilistic approach. Proc 31th Proc IEEE ICASSP vol 5 Toulouse France.

