# Supplementary figures and images for: Fast reproducible identification and large-scale databasing of individual functional cognitive networks
Source: BMC Neurosci. 2007 Oct 31;8:91. doi: 10.1186/1471-2202-8-91 (PMC2241626; doi:10.1186/1471-2202-8-91)

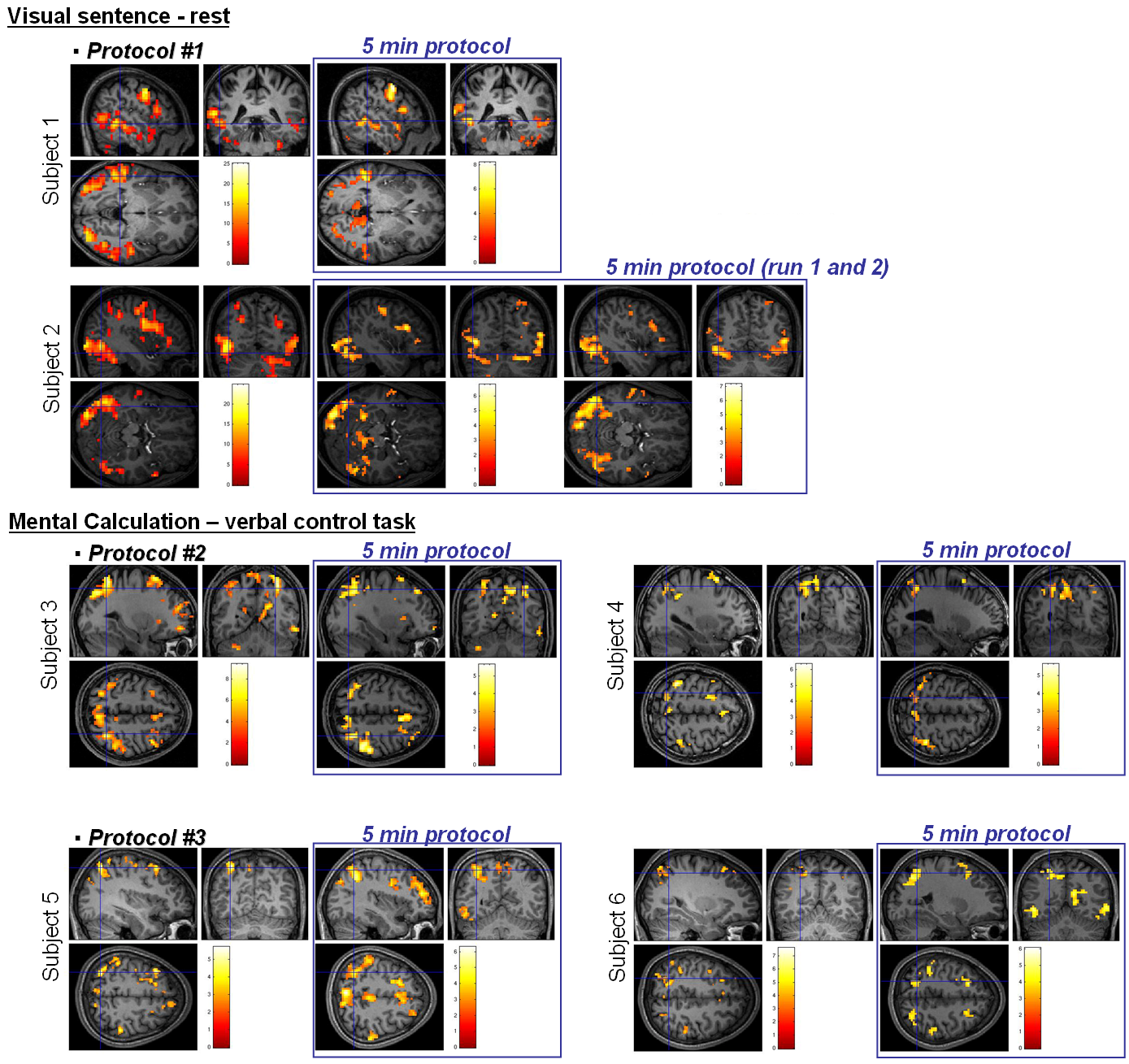

Supplement: Additional file 1 — Comparison of individual activation maps obtained from our 5 minutes protocol with maps computed from fMRI data acquired during the same session with another longer bloc design. As explained in the manuscript, our 5 minute protocol was added to each fMRI session performed in our laboratory. To estimate efficiency of our fast functional mapping and see if its results should be considered as free from its particular design, we compared some of our statistical maps with those corresponding to similar conditions performed in the associated main protocol (see details of each protocol below). We display here two examples of individual contrasts for three different fMRI protocols: one protocol including visual sentence reading (p = 0.001 corrected at the voxel level), and two other protocols including each a calculation task and a verbal control task (p = 0.001 uncorrected at the voxel level). Surrounded by a blue frames are reported a statistical map form the 5 min protocole for the corresponding subject at a similar anatomical location (p = 0.01 uncorrected at the voxel level, 30 voxels for cluster extent). Note that subject 2 performed twice our 5 min protocol with an interval of nine weeks, illustrating reliability of the activation topography. These maps suggest that even with quite different experimental conditions (block design versus fast event-related design, different control tasks, different rate of presentation, sometime different notation, different resolutions, different number of trials...) our 5 min design was able to capture most of the individual cerebral sites that characterize subject's functional activation during a task (here reading or calculating). In conclusion, maxima reported for each subject could be reasonably associated to areas that are crucial to perform a specified cognitive task, independently of the experimental conditions of stimulation and acquisition. Statistical thresholds were adapted for the obvious reason that the statistical power [file 1471-2202-8-91-S1.tiff]
