# Supplementary material for: Fast reproducible identification and large-scale databasing of individual functional cognitive networks
Source: BMC Neurosci. 2007 Oct 31;8:91. doi: 10.1186/1471-2202-8-91 (PMC2241626; doi:10.1186/1471-2202-8-91)
Supplement: Additional file 2 — List of Verbal stimuli. List of auditory and visually presented stimuli. Stimuli were presented in a French version, but translated for reader and reported here in an italic form. [file 1471-2202-8-91-S2.doc]

**Visual verbal Stimuli**

**Auditory verbal Stimuli**

***screen 1 screen 2 screen3 screen 4***

**▪ Motor task▪ Motor task**

appuyez trois fois sur le bouton droit appuyez trois fois sur le bouton droit

appuyez trois fois sur le bouton gauche appuyez trois fois sur le bouton gauche

*press three times on the right button press three times on the right button*

*press three times on the left button press three times on the left button*

l'orage a effrayé les animaux du zoo les gardiens du musée surveillent les tableaux

les chats guettent un oiseau sur le mur le samedi les magasins sont pleins de clients

le donjon du château tombe en ruine au cirques, les enfants on vu des tigres

du balcon on a vu le passage du défilé au japon les gens roulent a gauche

les ours adorent le saumon et le miel l'accident a fait de nombreuses victimes

en ville on trouve facilement des taxis on emprunte des livres a la bibliothèque

le froid de l'hiver a gelé le lac les pays chaud attirent les touristes

il y a beaucoup de ponts à Paris l'antenne de télé est fixée sur le toit

la pluie a rendu la route dangereuse un frigo permet de conserver les aliments

les roses sont belles mais elles piquent on appelle les pompiers en cas d'incendie

*the* *storm frightened the animals at the zoo the museum attendants watch the paintings*

*the cats are looking for a bird on the wall on sundays shops are full of clients*

*the keep of the castle falls into ruin children have seen tigers at the circus*

*we saw the march from the balcony in Japan, people drive on the left side*

*bears are fond of salmon and honey there were many victims from the accident*

*in town we easily find a taxi we could borrow books from the library*

*the cold of winter froze the lake warm countries attract tourists*

*there are many bridges in Paris the TV antenna is attached to the roof*

*the rain has made the road dangerous a fridge helps to preserve food*

*roses are nice but they prick we call for firemen in case of fire*

calculez seize moins huit calculez treize moins huit

calculez dix moins deux calculez onze moins cinq

calculez onze moins neuf calculez douze moins sept

calculez douze moins quatre calculez treize moins neuf

calculez dix-neuf moins six calculez dix-sept moins cinq

calculez seize moins deux calculez onze moins six

calculez treize moins sept calculez quinze moins huit

calculez dix-neuf moins sept calculez seize moins neuf

calculez onze moins trois calculez douze moins six

calculez dix-sept moins six calculez quatorze moins huit

*calculate sixteen minus eight calculate thirteen minus eight*

*calculate ten minus two calculate eleven minus five*

*calculate eleven minus nine calculate twelve minus seven*

*calculate twelve minus four calculate thirteen minus nine*

*calculate nineteen minus six calculate seventeen minus five*

*calculate sixteen minus two calculate eleven minus six*

*calculate thirteen minus seven calculate fifteen minus eight*

*calculate nineteen minus seven calculate sixteen minus nine*

*calculate eleven minus three calculate twelve minus six*

*calculate seventeen minus six calculate fourteen minus eight*

**▪ Reading task▪ Speech listening task**

**▪ Calculation task▪ Calculation task**
